# Supplementary material for: Norketamine, the Main Metabolite of Ketamine, Induces Mitochondria-Dependent and ER Stress-Triggered Apoptotic Death in Urothelial Cells via a Ca2+-Regulated ERK1/2-Activating Pathway
Source: Int J Mol Sci. 2022 Apr 23;23(9):4666. doi: 10.3390/ijms23094666 (PMC9102902; doi:10.3390/ijms23094666)
Supplement: Supplementary file 1 [file ijms-23-04666-s001.zip › ijms-1662967-supplementary.pdf]

Norketamine, the Main Metabolite of Ketamine, Induces Mitochondria-Dependent and ER Stress-Triggered Apoptotic Death in Urothelial Cells Via a Ca2+-Regulated ERK1/2-Activating Pathway

Jhe-Wei Lin, Yi-Chun Lin, Jui-Ming Liu, Shing-Hwa Liu, Kai-Min Fang, Ren-Jun Hsu, Chun-Fa Huang, Kai-Yao Chang, Kuan-I Lee, Kai-Chih Chang, Chin-Chuan Su and Ya-Wen Chen

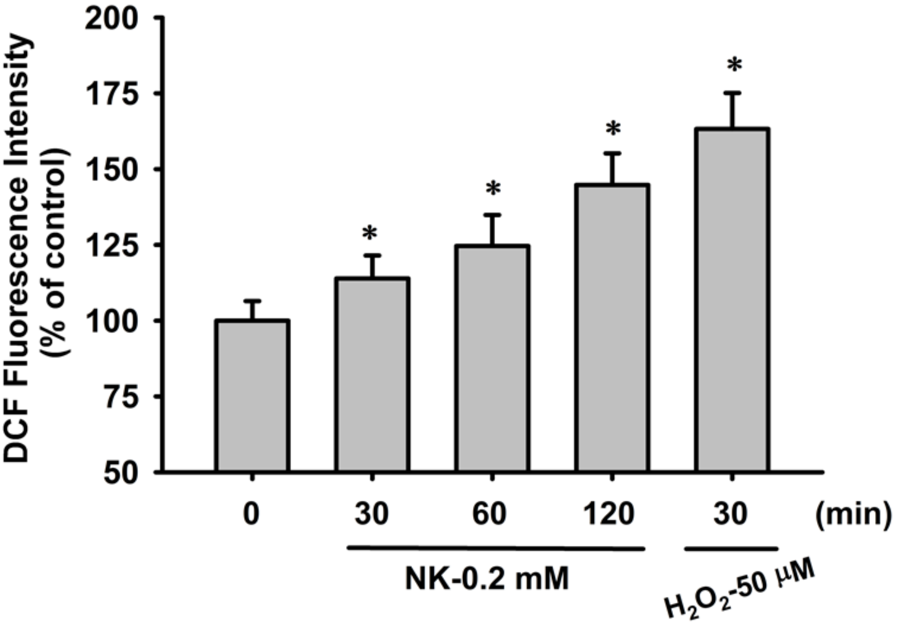

Figure S1. Effect of norketamine (NK) on ROS generation in RT4 cells. Cells were treated with NK (0.2 mM) for 30-120 min, and ROS generation was determined by flow cytometry using the fluorescent probe DCF. H_2O_2 (50 μM) was used as a positive control. Data are presented as mean ± SD of six independent experiments assayed in triplicate. **p* < 0.05 compared to vehicle control.

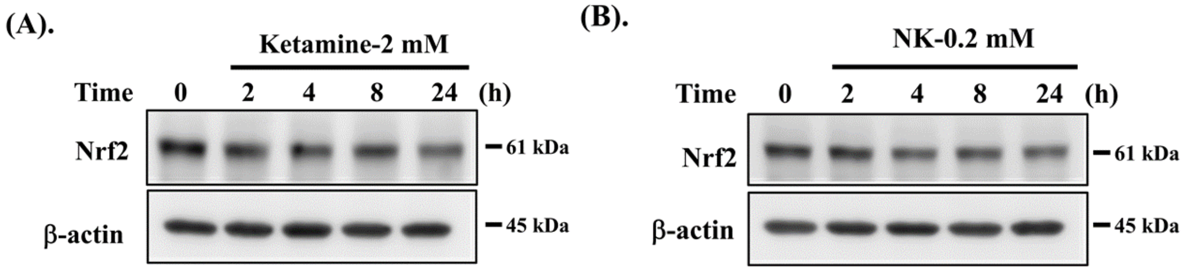

Figure S2. Effects of ketamine and norketamine (NK) on Nrf2 expression in RT4 cells. Cells were treated with (A) ketamine (2 mM) or (B) NK (0.2 mM) for various time intervals, and Nrf2 protein expression was examined using Western blot analysis. Results shown on a representative image, and β-actin was used as loading control.
